# Velocity-specific knee strength between professional and under-17 female volleyball players

**DOI:** 10.4102/sajp.v75i1.478

**Published:** 2019-06-11

**Authors:** Alexandre R.M. Pelegrinelli, Laís F. Dela Bela, Mariana F. Silva, Lucas C.R. Rodrigues, João P. Batista, Leandro C. Guenka, Josilainne M. Dias, Lee E. Brown, Rodrigo L. Carregaro, Felipe A. Moura, Jefferson R. Cardoso

**Affiliations:** 1 Laboratory of Biomechanics and Clinical Epidemiology, Research Group in Physical Therapy Assessment and Intervention (PAIFIT), Universidade Estadual de Londrina, Londrina, Brazil; 2 Laboratory of Applied Biomechanics, Universidade Estadual de Londrina, Londrina, Brazil; 3 Department of Geriatric Medicine, Uniklinik – Rheinisch–Westfälische Technische Hochschule (RWTH) Aachen University Hospital, Aachen, Germany; 4 Department of Kinesiology, California State University, Fullerton, United States; 5 School of Physical Therapy, Universidade de Brasília, Brasília, Brazil

**Keywords:** muscle strength, knee, dynamometer, volleyball, sports

## Abstract

**Background:**

Many studies have investigated isokinetic performance in volleyball players but not through surface maps.

**Objectives:**

The goals of this study were to assess velocity-specific isokinetic knee extensor–flexor muscle strength and to compare the isokinetic knee extensor–flexor muscles between professional (PRO) and under-17 (U17) female volleyball players.

**Method:**

This cross-sectional laboratory study was developed with two groups: PRO (*n* = 12), median_age_ = 21.3 years, and U17 (*n* = 9), median_age_ = 15 years. Peak torque, total work, mean power, angle of peak torque, hamstring–quadriceps torque ratio (H–Q ratio) and torque–angle–velocity surface maps were analysed from knee extension–flexion at 60, 120 and 300 degrees per second (°/s).

**Results:**

Significant differences were identified for extensor peak torque between PRO *x* = 202.3 Newton metre (N·m) (standard deviation [SD] = 24.4) and U17 *x* = 141.6 N·m (30.1) at 60 °/s (*p* < 0.001; *d* = 2.21) as well as flexor peak torque (PRO *x* = 75.7 N·m [10.3] and U17 *x* = 57.7 N·m [11.4]) at 120 °/s (*p* < 0.001; *d* = 1.65) for the dominant limb. There were also significant group differences for total work and mean power at all velocities for extension and flexion. Surface maps demonstrated higher torque at lower speeds for both groups with smaller torque changes across velocities for flexion.

**Conclusion:**

Different groups of female volleyball players showed contrasting concentric knee muscle strength across isokinetic velocities.

**Clinical implications:**

These results demonstrate the importance of specific strength training for different age groups, even within the same sport, and provide insight into muscle strength.

## Introduction

Volleyball is one of the most popular sports in the world. Within volleyball, vertical jumping is a crucial skill and important during blocking, attacking and net jousting (Lidor & Ziv 2010; Sattler et al. 2012). An elevated level of knee muscle strength is required during take-off and landing of a vertical jump, demanding high torque and power of concentric and eccentric actions of the knee extensor and flexor muscles (Dervisevic & Hadzic 2012).

Professionals and young athletes demonstrate distinct strength values because of different training volumes and musculoskeletal maturity (Bittencourt et al. 2005; Carling, Lee Gall & Malina 2012). During the career of a volleyball player, an increase in the intensity and frequency of training can be responsible for acute and chronic injuries and athletes dropping out of the sport (Solgird et al. 1995). These events can be explained by strength imbalances between muscle groups or between dominant and non-dominant limbs that predispose the joint to instability and ligament overload (Bamaç et al. 2008; Coombs & Garbutt 2002; Panni et al. 2002). Therefore, it is essential to quantify strength and identify agonist–antagonist muscle imbalances to reduce injury risk and provide training guidelines for muscle strength (Ardern et al. 2015; Dervisevic & Hadzic 2012; Hadzic et al. 2010).

Isokinetic dynamometry is a reliable method for evaluating muscle strength and is the gold standard to measure velocity-specific open kinetic chain torque, work and power, as well as muscle imbalances via agonist–antagonist torque ratios (Bamaç et al. 2008; Brown et al. 1993). Some previous studies have investigated isokinetic strength in professional volleyball players (D’Alessandro et al. 2005; Hadzic et al. 2010; Kabacinski et al. 2018; Sattler et al. 2015). A study by Bittencourt et al. (2005) compared under-19 and under-21 male players, and the authors found that the more experienced athletes had greater hamstring–quadriceps ratios and work for the knee flexors compared to the younger players. Furthermore, the importance of lower limb strength for these athletes is well recognised, and a broad analysis of velocity-specific strength is necessary for training (D’Alessandro et al. 2005; Panni et al. 2002).

Isokinetic strength assessments are mainly based on torque–angle representations; however, during dynamic assessments, the torque–angle relationship is affected by velocity increases (Frey-Law et al. 2012; Hewett, Myer & Zazulak 2008). Thus, three-dimensional (3D) surface maps have been recommended to visually represent the relationship between torque, angle and velocity and to characterise dynamic torque capabilities (Frey-Law et al. 2012; Khalaf et al. 2001). Finally, the analysis of muscle performance between age groups in female volleyball players may improve understanding about maturation for the sport and differences in training. The aims of this study were to assess velocity–specific isokinetic knee extensor–flexor muscle strength and to compare agonist–antagonist torque ratios and 3D surface maps between professional (PRO) and under-17 (U17) female volleyball players.

## Method

### Study design

This was a cross-sectional laboratory study.

### Study population

Twenty-one female city team volleyball athletes, who participated in the national championship, were divided into two groups: PRO (*n* = 12, median [Md][25% – 75%]) age 21.3 years [20–28.3], mass = 71.7 kg [67.2–79.3], height = 178.5 cm [169.2–184.0], body mass index [BMI] = 22.8 kg/cm^2^ [21.7–24.8]) with more than 8 years of training; and U17 (*n* = 9, Md age 15 years [14.5–16], mass = 56 kg [50.5–62.1], height =173.0 cm [165.5–178.0] and BMI = 19.3 kg/cm^2^ [18.3–20.1]) with approximately 3.5 years of training. Athletes were only included in the study if they reported no recent injuries and their frequency of training was 4–5 days per week. All assessments were performed at the beginning of the regular season.

### Data collection

Prior to testing, a 10-minute warm-up was performed on a cycle ergometer at a comfortable speed with no load. Next, assessments were conducted using a Biodex System 4 isokinetic dynamometer (Biodex Medical, Shirley, NY, USA), concentric mode, with the knee attachment. Participants were positioned sitting on a chair; the hip angle was set at 110 ° and the body stabilised with straps around the trunk, waist and thigh to avoid compensatory movements. The knee lateral condyle was the reference point for alignment with the machine’s rotation axis, and the lever arm was secured just above the medial malleolus (Brown & Weir 2001).

Range of motion (ROM) was set from 90 ° of knee flexion to 0 ° of extension. Gravity correction was performed with the lower limb relaxed at a 30 ° knee angle, according to the manufacturer’s manual. All tests were conducted by the same examiner and performed bilaterally at 60, 120 and 300 degrees per second (°/s), with a rest interval of 90 seconds between tests, starting with the dominant limb (leg used to kick a ball). A coefficient of variation less than 10% was used to ensure reliability of the test (Malina et al. 2005). Athletes were instructed to perform five full ROM maximal concentric knee extension–flexion repetitions with consistent verbal encouragement and visual feedback provided. They were encouraged to reach the mechanical ROM stops during both extension and flexion movements (Brown & Weir 2001). During the test, they were instructed to keep their arms crossed over their chest and not to move the contralateral leg. Prior to commencing the tests, all procedures were explained and practised as many times as necessary for familiarisation.

### Data analysis

To build 3D surface maps, isokinetic raw data were processed using a MATLAB^®^ algorithm (version R2009b, Math Works, Natick, MA, USA). Data were smoothed by a fourth-order Butterworth low-pass filter with cut-off frequencies of 20 hertz (Hz) for 60 °/s and 12 Hz for 300 °/s. All repetitions were used for analysis, and each was interpolated for each participant for all three velocities, in accordance with the duration and values of the average curves. To better characterise the surface maps, the entire ROM was considered, and the acceleration, constant velocity (load range) and deceleration phases of the movement were plotted. Surface maps are represented by Z-axis for torque, X-axis for angle and Y-axis for velocity, in which the darker (online: red) colour indicates the highest torque and the lighter (online: blue) colour the lowest torque. Peak torque ([PT], in Newton-metres [N·m]); total work ([TW], in joules [J]); mean power ([MP], in watts [W]) and angle of peak torque ([AngPT], in degrees [°]) were considered for analysis. Hamstring–quadriceps ratio (H–Q ratio) was only determined at 60 °/s as it is more sensitive to evaluation and indicates muscle imbalance (Lehnert, Xaverová & De Ste Croix 2014).

Demographic data normality was verified using the Shapiro–Wilk test, and the assumptions were not met; therefore, the values were presented as Md and quartiles (25% – 75%). To compare the H–Q ratio, an independent Student *t*-test was used, and the results are presented as mean and standard deviation. A generalised estimating equation model through a specific syntax was employed for comparisons within or between groups and velocities (60 °, 120 ° and 300 °/s). A working correlation matrix was specified a priori and defined the hypothesised relationship between repeated observations of a participant (model-based estimator). The model type was set as a linear scale response. The standard error estimates were adjusted according to the hypothesised correlation between different time points of the outcome (isokinetic data). The Bonferroni test for multiple comparisons was applied (Hanley et al. 2003). The effect size (*d*) was presented for the isokinetic variables in the three velocities. SPSS^®^ version 22 (IBM SPSS^®^, Armonk, NY, USA) was used for all analyses. Significance was set at 5%.

### Ethical considerations

The Universidade Estadual de Londrina Institutional Review Board approved the experiment (#055/2012) and all participants signed informed consent.

## Results

The variables were divided by muscle group, limb and age group. There were no statistically significant differences when comparing dominant versus non-dominant limbs. For knee extension, the PRO group demonstrated statistically significantly greater values with a large effect size for PT, TW and MP for all velocities, compared to U17 for the dominant limb. Similar results were found at all velocities for the non-dominant side, with no differences in AngPT for either side (Table 1).

**TABLE 1:** Isokinetic parameters of extensor muscle assessments.

Variable	60 °/s						120 °/s						300 °/s					
	PRO		U17		*p*	*d*	PRO		U17		*p*	*d*	PRO		U17		*p*	*d*
	Mean	SD	Mean	SD			Mean	SD	Mean	SD			Mean	SD	Mean	SD		
**Dominant**																		
Peak torque (N·m)	202.3	24.4	141.6	30.1	< 0.001	2.21	179.5	36.3	127.8	31.6	< 0.001	1.51	111.3	11.8	72.4	28.0	< 0.001	1.81
Total work (J)	1018.2	131.2	701.4	173.9	< 0.001	2.05	918.2	124.9	657.5	171.7	< 0.001	1.73	592.7	79.5	428.1	110.7	< 0.001	1.70
Mean power (W)	135.2	17.3	90.3	22.6	< 0.001	2.23	224.7	25.1	158.0	40.1	< 0.001	1.99	293.8	33.9	206.8	57.4	< 0.001	1.84
Angle of peak torque (°)	57.8	3.8	59.0	6.6	0.619	–0.22	53.0	3.8	54.8	5.3	0.338	–0.39	59.2	2.6	57.8	2.7	0.232	0.52
**Non-dominant**																		
Peak torque (N·m)	194.9	23.6	131.5	25.4	< 0.001	2.58	172.1	30.4	117.5	26.9	< 0.001	1.90	106.5	13.2	79.8	17.3	< 0.001	1.73
Total work (J)	961.0	108.3	648.2	141.9	< 0.001	2.47	860.8	116.7	605.7	146.8	< 0.001	1.92	562.8	81.2	412.5	105.4	< 0.001	1.59
Mean power (W)	128.5	15.1	84.5	17.5	< 0.001	2.69	210.8	27.2	146.5	34.1	< 0.001	2.08	276.0	39.1	191.3	42.8	< 0.001	2.06
Angle of peak torque (°)	58.5	4.7	57.5	9.8	0.779	0.13	55.6	4.7	56.6	6.4	0.679	–0.17	58.8	2.3	58.3	3.3	0.686	0.17

°/s, degrees per second; PRO, professional; U17, under 17; *d*, effect size; SD, standard deviation; N·m, Newton-metre; J, joule; W, Watt; °, degree.

For knee flexion of the dominant side, the PRO group demonstrated greater PT (120 °/s), with large effect sizes and TW and MP for all velocities, also with large effect sizes (Table 2). For the non-dominant side, PT (120 ° and 300 °/s), TW and MP (at all velocities) were statistically significantly greater for PRO athletes. No differences were observed in AngPT.

**TABLE 2:** Isokinetic parameters of flexor muscle assessments.

Variable	60 °/s						120 °/s						300 °/s					
	PRO		U17		*p*	*d*	PRO		U17		*p*	*d*	PRO		U17		*p*	*d*
	Mean	SD	Mean	SD			Mean	SD	Mean	SD			Mean	SD	Mean	SD		
**Dominant**																		
Peak torque (N·m)	91.5	12.3	77.1	30.5	0.157	0.61	75.7	10.3	57.7	11.4	< 0.001	1.65	77.8	12.9	69.1	15.9	0.159	0.60
Total work (J)	514.2	90.5	392.8	80.8	0.001	1.41	451.9	79.4	338.4	67.2	< 0.001	1.54	309.1	66.1	243.2	43.7	0.004	1.17
Mean power (W)	64.7	10.0	47.6	8.9	< 0.001	1.80	103.5	15.3	76.1	14.7	< 0.001	1.82	135.8	33.1	98.6	18.2	0.001	1.39
Angle of peak torque (°)	30.6	7.0	29.5	8.9	0.645	0.13	44.6	17.5	54.7	28.2	0.317	–0.43	86.5	1.5	87.6	1.7	0.093	–0.68
**Non-dominant**																		
Peak torque (N·m)	89.8	15.7	72.5	30.2	0.099	0.72	75.8	12.0	55.5	11.4	< 0.001	1.73	84.3	12.6	65.4	17.6	0.004	1.23
Total work (J)	516.4	108.1	367.3	70.6	< 0.001	1.63	455.3	89.2	324.2	66.7	< 0.001	1.66	312.3	64.1	227.2	48.7	< 0.001	1.49
Mean power (W)	64.7	11.6	45.1	8.6	< 0.001	1.91	102.8	17.3	71.9	15.2	< 0.001	1.89	172.2	29.6	88.2	19.8	< 0.001	3.33
Angle of peak torque (°)	34.1	13.8	36.2	18.3	0.763	–0.12	54.5	23.2	55.6	26.8	0.919	–0.04	86.4	0.9	79.1	27.5	0.399	0.37

°/s, degrees per second; PRO, professional; U17, under 17; *d*, effect size; SD, standard deviation; N·m, Newton-metre; J, joule; W, Watt; °, degree.

No difference was found for the H–Q ratio of the dominant limb (45.4% [SD = 4.5] for PRO and 50.2% [SD = 7.4] for U17). The non-dominant limb was 46.0% (SD = 5.0) for PRO and 50.5% (SD = 6.5) for U17.

**FIGURE 1:**
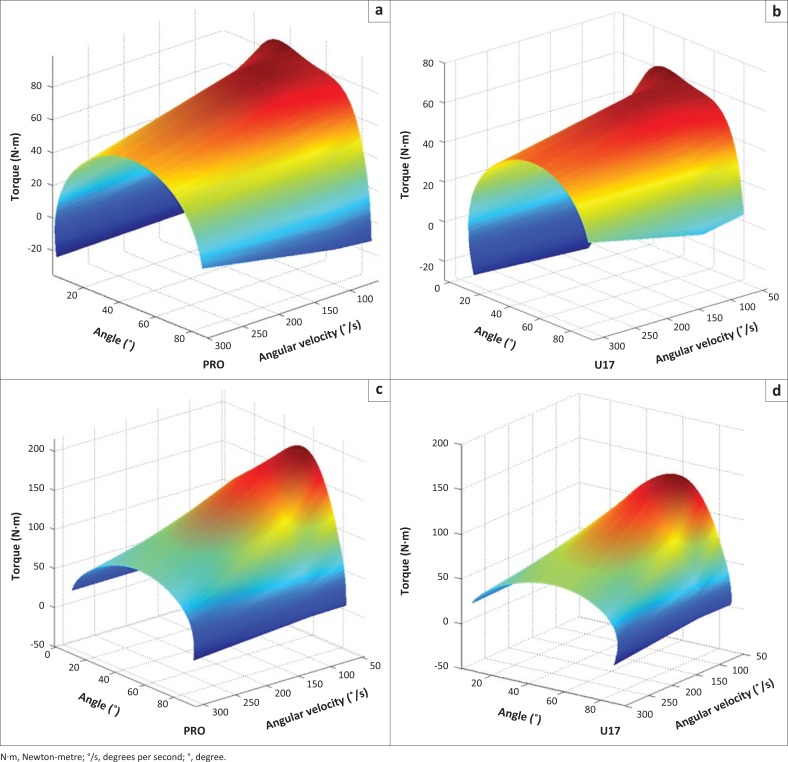
Three-dimensional surface maps of concentric contractions. (a) Knee flexion of the professional (PRO) group; (b) knee flexion of the under-17 (U17) group; (c) knee extension of the PRO group; (d) knee extension of the U17 group.

Because none of the variables demonstrated statistically significant differences between dominant and non-dominant limbs, 3D surface maps were only made for the dominant limb (Figure 1). The darker (red) area indicates higher extension torque for the PRO group. However, both groups demonstrated higher extensor torque values that were more concentrated at low and moderate speeds and produced a more acute ROM compared to the flexors. The darker (red) area also indicates higher flexion torque for the PRO group, while the U17 group showed less variation between speeds with a wider darker (red) area. In both groups, higher flexion torque values were concentrated at the end ROM compared to extension values.

## Discussion

The purpose of this study was to evaluate strength of the knee extensor and flexor muscles between two different age groups of female volleyball players. Isokinetic parameters were analysed for both the dominant and non-dominant limbs at low, medium and high speeds (60 °, 120 ° and 300 °/s, respectively) (Bittencourt et al. 2005; Dervisevic & Hadzic 2012; Hewett et al. 2008). Our findings demonstrated that the more experienced and biologically mature the player, the higher the values of PT, TW and MP at all velocities, for both extension and flexion movements. However, low H–Q ratios were found in both limbs, indicating possible muscle imbalances for both age groups.

Typically, professional players increase their capability to produce torque over years of training and, because of biological maturity, differences in strength become more evident between athletes of different ages (Carling et al. 2012; Hewett, Myer & Ford 2004). The results of our study support this and may contribute to understanding the influence of age on different isokinetic velocity specific strength measures.

The isokinetic parameters of PT, TW and MP were significantly higher for the PRO group than the U17 group for knee extension and flexion (knee flexors only at 120 °/s). Bittencourt et al. (2005) compared under-21 and under-19 volleyball players and found that the experienced athletes had higher PT and TW values during concentric knee flexion at 60 °/s. They concluded that this may be related to greater training volume in older athletes. Fousekis, Tsepis and Vagenas (2010) found that soccer players with more years of training produced higher torque while less experienced athletes presented greater muscle imbalances. These findings appear to be related to specificity of maturation, training frequency or volume. It is possible that musculoskeletal changes take place because of all these factors and that they are developed differently in each sport.

The H–Q ratios of both the PRO and U17 groups presented values between 45% and 50% for both limbs. Thus, considering that the reference value for a balanced joint reported in the literature for healthy people is approximately 60% (Coombs & Garbutt 2002), our results indicate potential knee muscle strength imbalances, especially for the PRO group. Previous studies comparing volleyball players of different age groups also demonstrated H–Q ratios below 60% for both limbs at 60 °/s and 300 °/s (Carling et al. 2012; Kabacinski et al. 2018). When comparing different sports, Magalhães et al. (2004) found an H–Q ratio of approximately 50% for volleyball players and 56% for soccer players at 90 °/s in both limbs. They suggested that volleyball players are at risk, and these low H–Q ratios may contribute to potential knee injuries. When elite volleyball and basketball players were compared, Bamaç et al. (2008) found H–Q ratios of 46% and 49% at 60 °/s. Based on these results and from a clinical perspective, decreases in the ability to decelerate knee extension (eccentric action of hamstring muscles) may result in an overload of the anterior cruciate ligament, thereby increasing joint instability and risk of injury (Kabacinski et al. 2018; Wilk et al. 1994).

In a volleyball match, jump performance has specific importance. During vertical jumps, the work of the knee muscles is responsible for 49% of the total work performed (Hubley & Wells 1983). Schons et al. (2018) found a reduced H–Q ratio in male volleyball players, and it was related to better performance in a countermovement jump. This may be associated with the need for greater generation of mechanical work for a jump, which is dependent on lower dissipation of the rotational energy between knee muscles (Prilutsky & Zatsiorsky 1994). Therefore, this difference between extensor and flexor muscles should contribute to jump performance.

Strength of the dominant and non-dominant limbs was also analysed in our study, with few differences between sides or groups. Presumably, the demands of running and jumping with both legs are higher than with one leg, leading to bilateral symmetry (Sattler et al. 2012, 2015). Additionally, previous investigations have concluded that isokinetic strength parameters are more related to vertical jumps than spike jumps and are associated with quadriceps concentric values (Sattler et al. 2015).

For knee flexion of both limbs, we found higher PT values at 300 °/s than at 120 °/s in both age groups. Peak torque is strongly influenced by acceleration and deceleration of the limb at high speeds during concentric actions (Iossifidou & Baltzopoulos 2000). These results illustrate the importance of representing the relationship between angle, torque and velocity with 3D surface maps. Surface maps visually display the behaviour of torque across velocities and provide qualitative feedback on the multiple biomechanical aspects of dynamic strength assessments (Khalaf et al. 2001). In our study, the interaction between angle and velocity on torque at 60 °/s was evident for the dominant limb while some differences were exhibited between age groups and muscles.

Extension torque presented a curvilinear map between joint angles and age groups. The majority of high torque values (darker or red area) were concentrated between 20 ° and 70 ° at low and moderate speeds (60 ° and 120 °/s), while at higher speeds torque dropped rapidly for the PRO group, with higher values more concentrated at a lower ROM (approximately 30 ° and 60 °). Despite the higher values achieved by the PRO group, the U17 group showed a darker (red) area changing smoothly at the other velocities, demonstrating maintenance of higher levels of torque at 300 °/s compared to PRO. At this high velocity, torque production appears to be more concentrated between 40 ° and 60 °.

Low torque values, indicated by lighter (blue) colour, were similar for both age groups, which is compatible with short muscle lengths during contraction. Low torque values in extension seem to not change considerably across velocities compared to high torque values. However, the light (blue) area represents acceleration and deceleration, and this may need to be taken into account (Iossifidou & Baltzopoulos 2000).

For the flexors, lower changes in high torque values were observed across velocities, primarily in the U17 group, demonstrated by a wider and steadier darker (red) area. The PRO group presented more variability in the darker (red) colour at higher velocities. However, the torque decline by velocity was quite linear and was similar between groups.

During knee flexion, PT occurred at various angles across velocities, while during extension, PT occurred consistently at approximately 60 °. According to Wretenberg et al. (1996), the flexor muscle moment arm varies with joint angle more than the extensors, and these variations are clearly visualised in the shape of the surface maps. Additionally, knee extensors have a mechanical advantage in the patella, which acts as a pulley mechanism across the femoral condyles and may help maintain the PT angle across velocities (Frey-Law et al. 2012).

Some limitations of our study include that during acceleration, some ROM was required to achieve the target velocity. Thus, this loss of isokinetic data might have influenced our results, especially at higher velocities. Although a constant hip position and stabilising straps were employed, compensatory movements may have influenced the test, as well as subsequent 3D surface maps. Frequency and intensity of training between age groups were also not controlled for.

### Recommendations

Future studies should attempt to determine the correlation between torque, angle and velocity in order to improve the analysis of strength between different age groups of volleyball players. The relationship between injury, H–Q ratio and jump performance should be explored in volleyball players. Additionally, an eccentric assessment would provide another perspective of strength, as this muscle action is often performed, especially with vertical jumps. Finally, surface electromyography of the agonist and antagonist muscles is highly recommended to identify muscle co-contraction across different velocities.

## Conclusions

Our findings demonstrate significantly greater knee extensor strength of PRO athletes when compared to U17. No significant differences were found for flexor PT at low and high velocities, while H–Q torque ratios demonstrated no differences between age groups but presented lower values than expected. Three-dimensional surface maps were able to illustrate the interaction between torque angle and torque velocity, as well as differences between age groups. Surface maps are an important tool to analyse torque variation across velocities and ROM, and they are recommended to help strength professionals better understand muscle performance.
